# Involvement of cAMP-Dependent Protein Kinase in the Nucleus Accumbens in Cocaine Versus Social Interaction Reward

**DOI:** 10.3390/ijms22010345

**Published:** 2020-12-31

**Authors:** Inês M. Amaral, Cristina Lemos, Isabella Cera, Georg Dechant, Alex Hofer, Rana El Rawas

**Affiliations:** 1 Division of Psychiatry I, Department of Psychiatry, Psychotherapy and Psychosomatics, Medical University Innsbruck, 6020 Innsbruck, Austria; ines.amaral@i-med.ac.at (I.M.A.); a.cristinalemos@gmail.com (C.L.); a.hofer@i-med.ac.at (A.H.); 2 Institute for Neuroscience, Medical University Innsbruck, 6020 Innsbruck, Austria; isabella_cera@libero.it (I.C.); georg.dechant@i-med.ac.at (G.D.)

**Keywords:** PKA, nucleus accumbens, cocaine, social interaction, reward, conditioned place preference, drugs, addiction, natural reinforcers, learning

## Abstract

Evidence suggests that PKA activity in the nucleus accumbens (NAc) plays an essential role in reward-related learning. In this study, we investigated whether PKA is differentially involved in the expression of learning produced by either natural reinforcers or psychostimulants. For that purpose, we inhibited PKA through a bilateral infusion of Rp-cAMPS, a specific PKA inhibitor, directly into the NAc. The effects of PKA inhibition in the NAc on the expression of concurrent conditioned place preference (CPP) for cocaine (drug) and social interaction (natural reward) in rats were evaluated. We found that PKA inhibition increased the expression of cocaine preference. This effect was not due to altered stress levels or decreased social reward. PKA inhibition did not affect the expression of natural reward as intra-NAc Rp-cAMPS infusion did not affect expression of social preference. When rats were trained to express cocaine or social interaction CPP and tested for eventual persisting preference 7 and 14 days after CPP expression, cocaine preference was persistent, but social preference was abolished after the first test. These results suggest that PKA in the NAc is involved in drug reward learning that might lead to addiction and that only drug, but not natural, reward is persistent.

## 1. Introduction

Natural reward behaviors and drug reward converge on a common neural pathway, the mesolimbic dopamine system, in which the nucleus accumbens (NAc) plays a central role [1]. Intracellular cascades may be differentially involved in the acquisition and expression of learning produced by either natural reinforcers or psychostimulant drugs [2]. More specifically, many studies demonstrate a role played by cyclic adenosine monophosphate (cAMP)-dependent protein kinase (PKA) in the NAc in reward-related learning [3,4]. For example, inhibition of PKA activity within the NAc has been reported to attenuate the acquisition of amphetamine [3] and the consolidation of cocaine-induced place conditioning [5], the acquisition of conditioned activity (CA) produced by NAc infusion of amphetamine [6], and cocaine-induced behavioral sensitization [7]. Furthermore, modulation of PKA has also been reported to alter drug self-administration behavior [8,9]. Together, these results provide evidence for the role of PKA in modulating reward-related learning and behavior that mediate responding for drugs [8]. However, the role of PKA in the NAc in modulating the expression of non-drug reward learning, such as social interaction (SI), has not yet been investigated.

Social interaction reward can be assessed using the conditioned place preference paradigm (CPP) with a weight-matched male conspecific [10,11]. Interestingly, physical contact—but not visual or olfactory cues—is the major rewarding sensory component of the composite stimulus “social interaction” [12,13]. When available as an alternative to drugs of abuse, social interaction rewards prevented reinstatement to cocaine [14,15,16], abolished cocaine preference [14,17], and eliminated drug self-administration, even in rats which met the criteria for addiction [18]. Furthermore, brain regions activated by the cocaine reward and the social interaction reward were shown to mostly overlap [19]. Although shared neuronal substrates may be needed for responding to natural rewards and drugs of abuse [20,21,22], it was hypothesized that intracellular pathways mediating natural rewarding stimuli are different from those involved in drug rewards.

The main scope of the study was to compare natural versus drug rewards. We focus on whether PKA in the NAc is differentially involved in the expression of learning produced by either social interaction or cocaine. Therefore, we first compared PKA activation levels in the NAc after cocaine or social interaction rewards. With the purpose of investigating the role of PKA in the NAc in the expression of reward-related learning of drug versus non-drug stimuli, we inhibited PKA through an infusion of Rp-cAMPS, a specific PKA inhibitor, directly into the NAc. The effects of PKA inhibition on the expression of concurrent CPP for cocaine and social interaction were subsequently investigated. For that, a concurrent paradigm [17,23,24] was used, where social interaction was made available in the alternative compartment associated with cocaine during conditioning. Therefore, a shift toward the cocaine- or social interaction-associated compartment could be indicative of the effects of intra-NAc PKA inhibition. In order to provide biochemical confirmation of Rp-cAMPS effects on PKA activity in the NAc, we measured the phosphorylation of two PKA substrates, cAMP response element-binding protein (CREB), and dopamine-regulated phosphoprotein-32 (DARPP-32) [9]. As positive social interaction is beneficial as an alternative to drugs [11], we evaluated whether the social interaction reward was continuous and compared the persistence of drug versus non-drug rewards. For that purpose, rats were trained to express cocaine or social interaction CPP and tested for eventual persisting preference 7 days and 14 days after CPP expression.

## 2. Results

### 2.1. Phosphorylated PKA (pPKA) Levels were Negatively Correlated with Cocaine Preference in the NAc

#### 2.1.1. Conditioned Place Preference for Cocaine or Social Interaction

**Figure 1:**
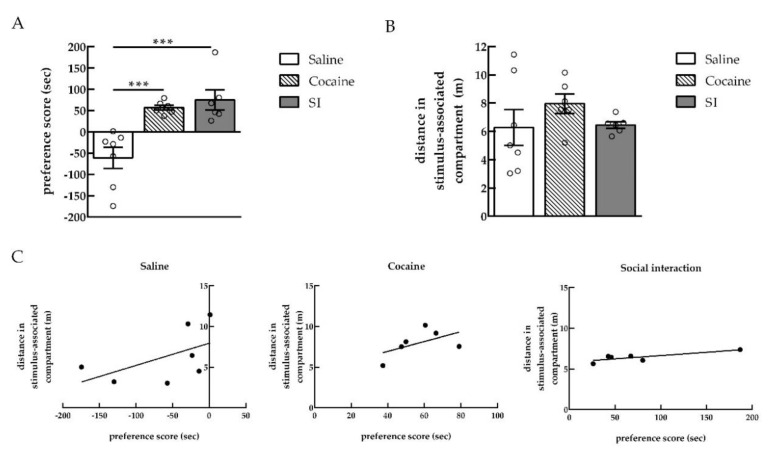
Cocaine and social interaction produced similar rewarding effects. (**A**) Conditioned place preference score after saline, cocaine, or social interaction (SI) conditioning (*n* = 6–7). (**B**) Distance traveled in the stimulus-associated compartment during the conditioned place preference (CPP) test (*n* = 6–7). (**C**) Correlation between conditioned place preference score and distance traveled in the stimulus-associated compartment during the test in saline, cocaine, and SI CPP groups (*n* = 6–7). *** p < 0.001, different from the saline CPP group, using a one-way ANOVA followed by Newman-Keuls multiple comparisons test.

Both cocaine (15 mg/kg) and social interaction with a conspecific of the same age and weight produced robust CPP, as evidenced by the higher preference score, when compared with rats from the saline CPP group which received saline i.p. injections in the two compartments of the CPP during the conditioning [one-way ANOVA, treatment effect, *F*_(2, 16)_ = 13.03; *p* = 0.0004; saline (*n* = 7) vs. cocaine (*n* = 6) *** p < 0.001, Cohen’s *d* = −2.47; saline vs. SI (*n* = 6) *** p < 0.001, Cohen’s *d* = −2.19]. Cocaine and social interaction produced a similar rewarding effect, as no significant difference between cocaine and social interaction CPP groups was found (Figure 1A).

Moreover, cocaine or social interaction CPP had no effect on the distance traveled in the stimulus-associated compartment when compared with saline CPP group [one-way ANOVA, treatment effect, *F*_(2, 16)_ = 1.010, *p* = 0.3864 (ns); saline (*n* = 7), cocaine (*n* = 6), SI (*n* = 6)] (Figure 1B). 

In order to establish a possible correlation between the preference score and the distance traveled in the stimulus-associated compartment, the Pearson correlation coefficient was calculated. Whereas cocaine preference was not correlated with the distance in the cocaine-associated compartment, a trend for a positive correlation between social preference and the distance in the compartment associated to social conditioning was observed [Pearson correlation coefficient; saline (*n* = 7): *r* = 0.54, *p* = 0.2136 (ns); cocaine (*n* = 6): *r* = 0.54, *p* = 0.2745 (ns); SI (*n* = 6): *r* = 0.81, *p* = 0.0508] (Figure 1C).

#### 2.1.2. PKA Expression in the NAc after Cocaine or Social Interaction Preference

**Figure 2:**
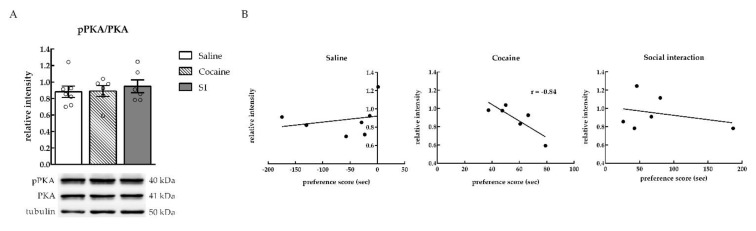
pPKA levels in the nucleus accumbens (NAc) were negatively correlated with cocaine preference. (**A**) Relative intensity of phosphorylated PKA (pPKA) in saline, cocaine, and SI CPP groups. (*n* = 6–7). Representative Western blot images are shown below the graphs. (**B**) Correlations between preference score and relative intensity of pPKA in the NAc of saline, cocaine, and SI CPP groups (*n* = 6–7).

Twenty minutes after the CPP test, phosphorylation levels of PKA were not changed in the NAc of animals that expressed cocaine or SI CPP in comparison to the saline group [one-way ANOVA, Treatment effect, *F*_(2, 16)_ = 0.2590; *p* = 0.7750 (ns); saline (*n* = 7), cocaine (*n* = 6), SI (*n* = 6)] (Figure 2A). However, a negative correlation was found between the preference score and the relative intensity of pPKA in the cocaine CPP group. Indeed, rats with a lower cocaine preference score showed higher levels of PKA activation [Pearson correlation coefficient; saline (*n* = 7): *r* = 0.24, *p* = 0.6033 (ns); cocaine (*n* = 6): *r* = −0.84, *p* = 0.0384 (*); SI (*n* = 6): −0.30, *p* = 0.5751 (ns)] (Figure 2B).

### 2.2. PKA Inhibition in the NAc Shifted the Preference Toward Cocaine

**Figure 3:**
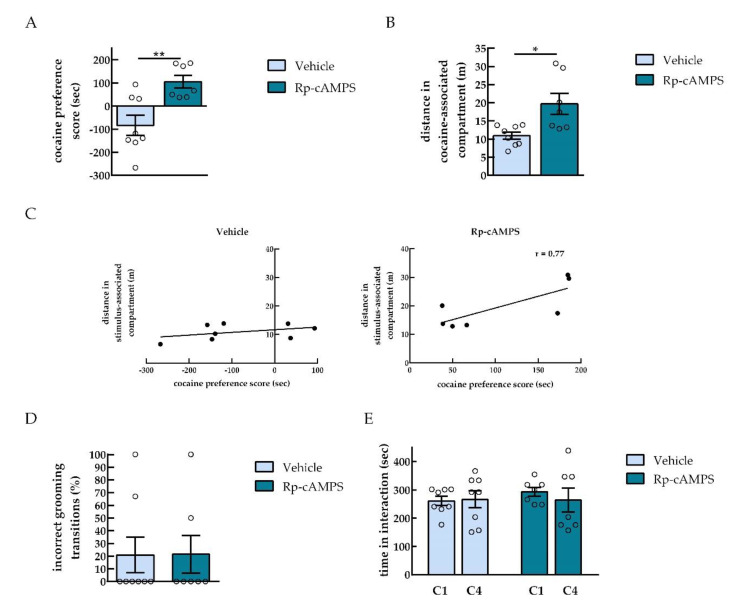
PKA inhibition in the NAc shifted the preference toward cocaine. (**A**) Cocaine preference score of rats infused with vehicle or PKA inhibitor (Rp-cAMPS) and exposed to both cocaine and social interaction in a concurrent CPP paradigm (*n* = 7–8). (**B**) Distance traveled in the compartment paired with cocaine, during the CPP test (*n* = 7–8). (**C**) Positive correlation between cocaine preference and the distance in the cocaine-associated compartment during the test, following PKA inhibition (*n* = 7–8). (**D**) Percentage of incorrect transitions of cephalocaudal grooming progression during the CPP test in rats infused with vehicle or Rp-cAMPS (*n* = 7–8). (**E**) Time spent in social interaction during the first (C1) and last (C4) social interaction conditioning session (*n* = 7–8). ** p < 0.01, using an unpaired *t*-test, different from vehicle; * p < 0.05, using an unpaired *t*-test with Welch’s correction, different from vehicle.

In order to investigate the effects of intra-NAc inhibition of PKA on cocaine and SI CPP expression, we infused the cAMP analog Rp-cAMPS in the NAc of rats conditioned to both cocaine and social interaction in a concurrent CPP paradigm. As expected [14,17], rats conditioned simultaneously with cocaine in one compartment and SI in the other compartment expressed no preference for cocaine (Figure 3A). However, PKA inhibition in the NAc shifted the preference toward cocaine as compared to the vehicle group [two tailed unpaired *t*-test, ** p < 0.01 (*p* = 0.0036), *t* = 3.54, *df* = 13; Cohen’s *d* = −1.87; *n* = 7–8] (Figure 3A). The infusion of Rp-cAMPS also increased the locomotor activity of the rats in the cocaine-paired compartment when compared to the vehicle group [two tailed unpaired *t*-test with Welch’s correction, * p < 0.05 (*p* = 0.0227), *t* = 2.87, *df* = 7.404; Cohen’s *d* = −1.49; *n* = 7–8] (Figure 3B). Furthermore, this distance was positively correlated with cocaine preference after infusion with Rp-cAMPS in the NAc [Pearson correlation coefficient; vehicle (*n* = 8): *r* = 0.41, *p* = 0.3128 (ns); Rp-cAMPS (*n* = 7): *r* = 0.77, *p* = 0.0437 (*)] (Figure 3C). 

To check for any effects of PKA inhibition on the stress levels of rats, which may result in increased cocaine preference, the percentage of incorrect grooming pattern progression during the CPP test, used as a stress marker in rodents, was evaluated. The infusion of Rp-cAMPS did not affect the incorrect transition of grooming behavior when compared with vehicle-infused rats [unpaired *t*-test, *p* = 0.9788 (ns), *t* = 0.027, *df* = 13, *n* = 7–8] (Figure 3D), meaning that the stress levels of vehicle- and Rp-cAMPS-infused rats are similar. 

In order to investigate whether increased cocaine preference was due to less social interaction between paired rats, time spent in direct social contact during the first and last social interaction conditioning session was also quantified. Time in direct contact between paired rats during the first (C1) and last (C4) social conditioning sessions was not different following infusions with vehicle or Rp-cAMPS [two-way ANOVA, treatment effect, *F*_(1, 13)_ = 0.4286, *p* = 0.5241; conditioning session effect, *F*_(1, 13)_ = 0.1443, *p* = 0.7102; treatment × conditioning session interaction, *F*_(1, 13)_ = 0.3052, *p* = 0.59, *n* = 7–8] (Figure 3E), suggesting that Rp-cAMPS infusion did not affect social contact in paired rats. 

### 2.3. NAc Infusion of Rp-cAMPS Reduced Levels of DARPP-32 Phosphorylation

**Figure 4:**
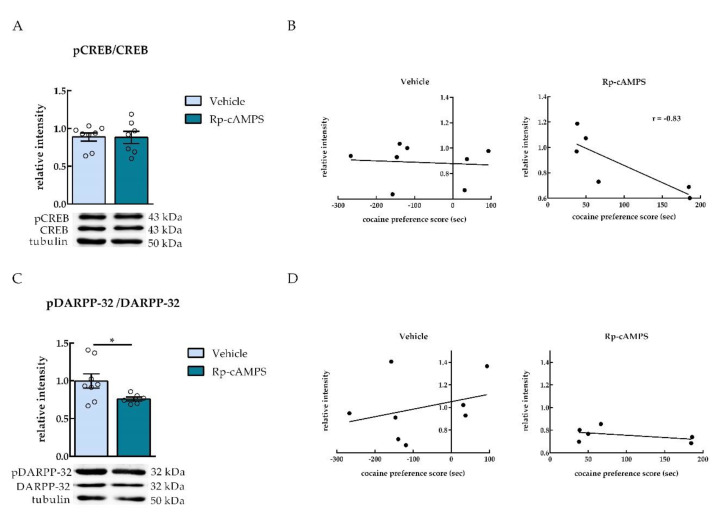
Infusions of Rp-cAMPS reduced levels of pDARPP-32 in the NAc. (**A**,**C**) Relative intensity of (**A**) phosphorylated CREB (pCREB) and (**C**) phosphorylated DARPP-32 (pDARPP-32) in the NAc of animals infused with either vehicle or the PKA inhibitor Rp-cAMPS (*n* = 6–8). Representative Western blot images are shown below the graphs. (**B**,**D**) Correlation between cocaine preference score and relative intensity of pCREB (**B**) and pDARPP-32 (**D**) in the nucleus accumbens of vehicle and Rp-cAMPS-infused animals (*n* = 6–8). * p < 0.05, using an unpaired *t*-test with Welch’s correction, different from vehicle.

In order to provide biochemical confirmation of Rp-cAMPS effects on PKA activity in the NAc, we measured the phosphorylation of a well-characterized PKA substrate, CREB. Unexpectedly, no differences were detected in the relative intensity of phosphorylated CREB (pCREB) in the NAc between the vehicle and Rp-cAMPS groups (unpaired *t*-test, *p* = 0.9507 (ns), *t* = 0.06304, *df* = 13, *n* = 7–8) (Figure 4A). However, a negative correlation was found between cocaine preference and the relative intensity of pCREB in the group of animals infused with Rp-cAMPS. In fact, rats with lower cocaine preference scores presented higher CREB phosphorylation levels [Pearson correlation coefficient; vehicle (*n* = 8): *r* = −0.09, *p* = 0.8343 (ns); Rp-cAMPS (*n* = 6): *r* = −0.83, *p* = 0.0433 (*)] (Figure 4B). Another well-characterized PKA substrate is DARPP-32, which is activated by PKA upon phosphorylation in the threonine 34 [9]. The levels of phosphorylated DARPP-32 were significantly reduced in the NAc of Rp-cAMPS compared to the vehicle-infused group of rats [two tailed unpaired *t*-test with Welch’s correction, *p* = 0.0417, *t* = 2.420, *df* = 8.040, Cohen’s *d* = 1.22, *n* = 6–8] (Figure 4C). No correlations were found between cocaine preference and pDARPP-32 relative levels in the NAc of both vehicle and Rp-cAMPS groups [Pearson correlation coefficient; vehicle (*n*= 8): *r* = 0.31, *p* = 0.4613 (ns); Rp-cAMPS (*n* = 6): *r* = −0.45, *p* = 0.3659 (ns)] (Figure 4D). 

### 2.4. PKA Inhibition in the NAc did not Affect Social Interaction Reward

**Figure 5:**
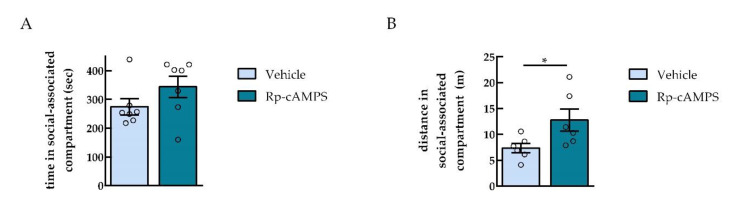
PKA inhibition in the NAc had no effect on social interaction CPP. (**A**) Time animals spent in the social interaction-associated compartment during the CPP test. (**B**) Distance traveled in the social interaction-associated compartment during the CPP test. * p < 0.05, using an unpaired *t*-test, different from vehicle.

In order to study the effects of PKA inhibition in the NAc on social interaction CPP expression, Rp-cAMPS was infused before the CPP test to rats previously conditioned to saline and social interaction. The infusion of Rp-cAMPS did not change the time rats spent in the social interaction-paired compartment during the CPP test compared to the vehicle-infused group [two-tailed unpaired *t*-test, *p* = 0.1630 (ns), *t* = 1.486, *df* = 12, *n* = 7] (Figure 5A). Additionally, when calculating the social preference score of the Rp-cAMPS-infused group, no differences were observed when comparing the vehicle to the Rp-cAMPS group [vehicle group: 58 ± 40 sec vs. Rp-cAMPS group: 95 ± 23 sec, two-tailed unpaired *t*-test, *p* = 0.4388 (ns), *t* = 0.8009, *df* = 12; *n* = 7]. Moreover, the social preference score of the vehicle group was not different from the group “without surgery” conditioned with social interaction in Figure 1A [vehicle group: 58 ± 40 sec vs. “without surgery” group: 75 ± 24 sec, two-tailed unpaired *t*-test, *p* = 0.7354 (ns), *t* = 0.3467, *df* = 11; *n* = 6–7]. In line with the observations in the concurrent CPP paradigm (Figure 3B), Rp-cAMPS infusion significantly increased the distance animals traveled in the social interaction-associated compartment (two-tailed unpaired *t*-test, * p < 0.05 (*p* = 0.0421), *t* = 2.330, *df* = 10; Cohen’s *d* = −1.58; *n* = 6).

### 2.5. Persistence of Drug vs. Behavior Non-drug Conditioned Place Preference

**Figure 6:**
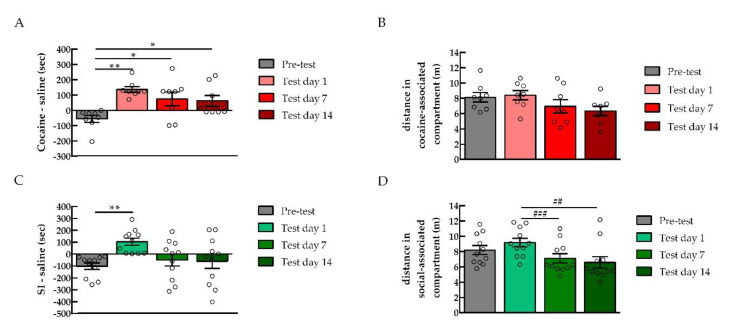
Preference for cocaine is persistent over a period of 14 days after the CPP testing, whereas preference for social interaction is lost over time. (**A**) Preference for cocaine, presented as time spent in the cocaine-associated compartment minus the time spent in the saline compartment, in the pre-test, the first CPP test (test day 1), second (test day 7), and third (test day 14) tests (*n* = 8). (**B**) Distance traveled in the stimulus-associated compartment during the pretest and CPP tests (*n* = 8). (**C**) Preference for social interaction (SI), presented as time spent in the SI-associated compartment subtracted by time spent in the other (saline) compartment, in the pre-test, test day 1, test day 7, and test day 14 (*n* = 11). (**D**) Distance traveled in the stimulus-associated compartment in the pretest and following tests performed (*n* = 11). * p < 0.05 and ** p < 0.01, repeated measures one-way ANOVA, compared to pre-test. *## p* < 0.01 and *### p* < 0.001, repeated measures one-way ANOVA, compared to test day 1.

As previously shown [14], cocaine CPP can persist up to several days after the last conditioning session. A significant preference for the cocaine-paired compartment was observed in rats subjected to cocaine CPP [Repeated measures one-way ANOVA, test day effect, *F* = 7.703; *p* = 0.0046, *n* = 8]. On test day 1, rats conditioned to cocaine demonstrated a strong preference for the stimulus-associated compartment [Pre-test vs. Test day 1 (*n* = 8), ** p < 0.01, Cohen´s *d* = −3.28] as shown by the difference between the time animals spent in the cocaine-paired compartment and the time they spent in the saline-associated compartment. This preference persisted on test day 7 [Pre-test vs. Test day 7 (*n* = 8), * p < 0.05, Cohen´s *d* = −1.29] and test day 14 [Pre-test vs. Test day 14 (*n* = 8), * p < 0.05, Cohen´s *d* = 1.39] (Figure 6A). The locomotor activity of the animals in the cocaine-paired compartment was not different between the test days (Figure 6B). In another set of experiments, other rats were conditioned to social interaction and, on test day 1, a significant preference for the SI-paired compartment was observed [Repeated measures one-way ANOVA, test day effect, *F* = 5.258; *p* = 0.0159; *n* = 11; Pre-test (*n* = 11) vs. Test day 1 (*n* = 11) ** p < 0.01, Cohen´s *d* = −2.20]. Contrary to cocaine CPP, when tested on day 7 after the first test, the initial preference of these rats for the SI-associated compartment was not expressed anymore [Pre-test vs. Test day 7 (*n* = 11), *p* > 0.05]. The same effect was observed on test day 14 [Pre-test vs. Test day 14 (*n* = 11), *p* > 0.05] (Figure 6C). Rats conditioned to social interaction showed a decrease in the locomotor activity associated with the social-compartment during test day 7 and test day 14 compared to test day 1 [Repeated measures one-way ANOVA, test day effect, *F* = 12.33; *p* = 0.0005; *n* = 11; Test day 1 vs. Test day 7, *### p* < 0.001, Cohen´s *d* = 1; Test day 1 vs. Test day 14, *## p* < 0.01, Cohen´s *d* = 0.78; Test day 7 vs. Test day 14, *p* > 0.05] (Figure 6D).

## 3. Discussion

The main finding of this study is that inhibition of PKA in the NAc increased the expression of cocaine preference. This effect was not due to increased stress levels as evaluated by incorrect cephalocaudal transitions used as a marker of stress, or a difference in social interaction reward in the Rp-cAMPS-inhibited group. Moreover, whereas drug-rewarding properties are persistent and long lasting, non-drug rewards such as social interaction are lost over time. Therefore, when used as an alternative to drugs of abuse, social interaction should be maintained continuously in order to be effective.

Several studies have reported that the repeated administration of cocaine and other psychostimulants can increase activity and/or levels of PKA [25,26,27,28]. Our results show that cocaine CPP did not induce PKA activity in the NAc in comparison with social interaction CPP and saline control animals. Rather, in the cocaine group, a negative correlation was found between pPKA levels and cocaine preference, reflecting that a lower cocaine preference was associated with higher pPKA levels in the NAc. The present findings indicate that PKA might exert a «tonic» inhibitory effect on the expression of cocaine preference at low-dose regimen. In fact, it appears that chronic and intensive cocaine dose regimes like those employed by [25,26] are required for cocaine-induced increases in PKA activity. In Terwilliger et al.’s [26] study, a prolonged drug-exposure regimen (i.e., 14 consecutive days of twice-daily injections of 15 mg/kg cocaine) increased PKA activity in the NAc. Moreover, Lu et al. [25] examined PKA activity at several different cocaine abstinence periods after high-dose cocaine self-administration. Indeed, in the accumbens of cocaine-trained rats, PKA activity levels were increased on days 1 and 30, but not on day 90 of reward withdrawal [25]. Consistent with this interpretation, Unterwald et al. [29] reported that an intensive 14-day cocaine regimen (45 mg/kg/day), but not a 1- or 7-day regimen, enhanced adenylyl cyclase activity in the NAc and caudate putamen. In line with the latter study, when a less intensive cocaine regimen (i.e., 7 consecutive days of once-daily injections) was used, a significant decline in accumbal PKA activity was observed rather than an increase [30]. Therefore, it is plausible that in the present study, a daily dose of 15 mg/kg of cocaine (i.e., 4 consecutive days of once-daily injections) did not induce an increase in PKA activity levels in the NAc. Social interaction CPP as assessed in our study did not alter PKA activity in the NAc, suggesting that pPKA levels seems not to be affected by non-drug reward learning. 

The present findings are consistent with the previous literature implicating PKA in the NAc in reward-related learning and incentive motivation for rewards [6,7,8,9,31]. Our results show that PKA inhibition in the NAc produced an increase in cocaine preference expression. In a model of psychostimulant drug conditioning, the expression of CA produced by NAc injections of amphetamine was also enhanced by PKA inhibition [2]. Consistent with the observed enhancement, the expression of lever pressing for cocaine was also increased by NAc PKA inhibition with Rp-cAMPS [9]. Indeed, it seems that PKA inhibition in the NAc impairs the acquisition of conditioned place preference and the CA induced by NAc injections of amphetamine [3,6], but not the expression of amphetamine-produced conditioning [2]. However, ICV infusions of H7, a non-selective inhibitor of protein kinases, significantly reduced the time spent in the cocaine compartment when given immediately after each conditioning session (consolidation), whereas it had no such effect when administered before cocaine during the training phase (acquisition) or before testing for place preference in the absence of cocaine (expression) [5]. These results suggest that changes in the activity of PKA, together with protein kinase C [5] during the consolidation phase of cocaine CPP, might affect drug reward learning. 

When using the concurrent paradigm by pairing cocaine with one compartment and social interaction with the alternative compartment of the CPP, our results showed an increase in cocaine preference after intra-NAc PKA inhibition engender three different possibilities. The first possibility is that PKA inhibition by Rp-cAMPS increased the rewarding effects of cocaine consistently with the findings of [2,9]. The second possibility is that the observed increase in cocaine preference might result from altered stress levels in rats infused by Rp-cAMPS. Indeed, interactions between stress and PKA signaling have previously been reported [32,33], with stress known to promote the effects of drugs [17,34]. For that, we evaluated the percentage of incorrect cephalocaudal transitions [17] considered as a marker of stress [35] in animals infused with Rp-cAMPS or vehicle. Incorrect grooming transitions were not different between the two groups, suggesting that rats infused with Rp-cAMPS and vehicle have similar levels of stress. The third possibility is that the enhancement in cocaine preference in the Rp-cAMPS group is the result of a decrease in social interaction. In order to investigate this possibility, considering that “touch” is the most rewarding component in social interaction reward [12], we assessed the time during which the rats got in physical contact during the first (C1) and the last (C4) conditioning. Time of direct social interaction during the first or the last conditioning sessions was not different between the vehicle and the Rp-cAMPS groups. Together, these findings suggest that the increase in cocaine preference in the Rp-cAMPS group was not the result of enhanced stress levels or an impairment in social interaction. In agreement with the study by Kummer et al. [36], the time in direct contact remained unchanged between conditioning sessions C1 and C4. However, the time rats spent in direct contact in the social interaction conditioning sessions in this study seems to be lower than the time in interaction evaluated in a previous study by Kummer et al. [36]. Yet, this could simply be due to the manual assessment of this “time in contact” by two different experimenters. More importantly, social interaction in this study (Figure 3A vehicle group), as well as in [36], similarly abolished the preference for cocaine (15 mg/kg) when available in the alternative context in a concurrent CPP paradigm. Therefore, the fact that rats developed equal preference to social interaction and cocaine in a concurrent CPP confirms our previous findings, showing that social interaction and cocaine (15 mg/kg) in rats have the same reward value [14,17,24,36]. 

In order to investigate the effects on PKA activity in the NAc after Rp-cAMPS infusions, we measured the phosphorylation state of two well-characterized PKA substrates, CREB and DARPP-32 [9]. Unexpectedly, the levels of pCREB were not changed between the Rp-cAMPS- and vehicle-infused rats, but rather a negative correlation was found between pCREB levels in the NAc and cocaine preference. This correlation reflected that lower levels of pCREB in the NAc were linked to higher preference scores to cocaine. Interestingly, levels of pDARPP-32 were significantly lower in the NAc of Rp-cAMPS-infused rats as compared to the vehicle group. Although the effects of Rp-cAMPS infusions on cocaine preference may not with all certainty involve DARPP-32 or CREB, these PKA substrates demonstrate at least the PKA inhibition in the NAc.

The NAc can be subdivided into core and shell regions and a substantial amount of data indicates that these sub-regions may be differentially involved in reward-related learning [37,38,39,40]. In a concurrent paradigm in which rats were conditioned to cocaine or social interaction, a pre-acquisition lesion of the NAc core or the basolateral amygdala shifted the animals’ preference toward social interaction CPP, whereas a bilateral NAc shell lesion shifted the preference toward cocaine CPP [24]. These findings suggest a role of the NAc shell in mediating social interaction reward, and a role of the NAc core and the basolateral amygdala in mediating cocaine reward [24]. Furthermore, the correlations of the activation of the NAc core and NAc shell sub-regions with the time spent in the cocaine- or the social interaction-associated compartment, respectively, at the expression time point of CPP has previously been described [19]. In the present study, all infusion sites that were aimed into the NAc mainly targeted the core of the NAc sub-region, which is principally involved in drug reward [24]. Yet, it appears that inhibiting PKA with Rp-cAMPS mainly in the core sub-region of the NAc enhanced the expression of lever-pressing for cocaine [9] and cocaine preference (present study).

In order to determine the effects of Rp-cAMPS on natural reward expression, another group of rats conditioned only with social interaction received an intra-NAc Rp-cAMPS infusion before the CPP test. Our results show that the Rp-cAMPS group developed a social interaction preference similar to the vehicle group. These results suggest that the inhibition of PKA in the NAc did not alter the expression of non-drug reward learning. In line with these findings, behavioral work has implicated PKA in the acquisition, but not the expression of lever pressing for food [9,31] or sucrose intake [41]. These observations further support our findings in the concurrent paradigm following PKA inhibition that the enhancement in cocaine preference did not result from a reduction in social interaction rewards. 

There was a dissociation between the rewarding and the locomotor effects of cocaine (Figure 1B). Indeed, although cocaine produced a preference to the cocaine-associated compartment, the distance traveled by the rats was not different between the saline control and cocaine-conditioned rats. It appears that the rewarding properties of the drugs are not dependent on locomotor stimulation [42]. Similarly, rats tested for cocaine persistence still showed preference for the cocaine-associated compartment with no changes in the locomotor activity across the test days (Figure 6B). However, when social interaction preference was lost 7 and 14 days after the CPP test, the distance rats traveled in the social-associated compartment was also reduced. This loss of preference might reflect a reduced interest in exploring the social-associated context. Yet, similarly to drugs, non-drug rewarding properties appear to be independent of locomotor stimulation. Indeed, rats that expressed social preference in (Figure 1B) did not show an increase in locomotor counts compared to saline control animals.

In the CA paradigm based on NAc injections of amphetamine, the groups that were conditioned with amphetamine and tested after Rp-cAMPS infusion showed higher activity than the group conditioned with amphetamine and tested with saline [2]. Interestingly, PKA inhibition on test day had no effect on activity for rats conditioned with vehicle, suggesting that Rp-cAMPS had no locomotor effects on its own [2]. In line with these observations, in this study rats that were infused with Rp-cAMPS showed higher locomotor activity in the cocaine-associated compartment compared to the vehicle group. Furthermore, a positive correlation was found between the distance associated with the drug-related compartment and cocaine preference. Therefore, exposure to the drug-associated compartment during the CPP test likely enhanced dopamine release in a comparable way to the exposure to the amphetamine associated box on test day [2]. It was suggested that Rp-cAMPS can enhance locomotion only in the presence of increased ongoing dopamine activity [2]. Despite a lack of difference in social preference expression between the vehicle and the Rp-cAMPS group, PKA inhibitor infusion before the CPP test also increased the locomotor activity of rats in the social-associated compartment during the CPP test. Thus, exposure to non-drug rewarding stimuli might also have caused dopamine release to a level which could promote locomotor activity by Rp-cAMPS. 

Several studies reported that drug-induced CPP is persistent [14,43]. Our results show that rats still expressed cocaine preference when tested 14 days after the end of CPP training. However, rats conditioned to social interaction lost their preference to the social stimulus-associated compartment in the tests that occurred 7 days and 14 days after the first CPP test. These results suggest that, whereas drug reward is long-lasting, non-drug reward is not persistent. In line with these findings, previous work has shown that prevention by social interaction against cocaine relapse is ineffective if not maintained continuously in a drug-free environment [15], most likely because social interaction effects are not persistent. 

The present behavioral results provide evidence for the involvement of PKA in the NAc in drug reward learning that might lead to addiction. However, the expression of learning for natural reward does not appear to be under the control of PKA. In line with previous findings reporting that conditioning with social interaction must be long enough to protect against subsequent exposure to cocaine [15], this data shows that non-drug reward is not persistent. In order to promote resilience against stress and drugs of abuse [11], prevention by social interaction should therefore be maintained continuously in a drug-free context. 

## 4. Materials and Methods 

### 4.1. Animals 

Male Sprague-Dawley rats aged 6–7 weeks (150–250 g) were obtained from Janvier Labs, France. The animals were housed at a constant room temperature of 24 °C and had ad libitum access to water and pellet chow. Experiments were performed during the light phase of a continuous 12 h-light/dark cycle. Animals were isolated upon arrival to the animal facility, habituated to handling for 5 min for a period of three days before the start of the behavioral experiments or surgeries, and remained singly housed during the entire experiment. All animals were 8-weeks old when subjected to experimental behavior. All experiments were approved by the Austrian National Animal Experiment Ethics Committee, permit numbers BMWF-66.011/0131-WF/V/3b/2016 and BMWF-66.011/0040-WF/V/3b/2019. 

### 4.2. Place Conditioning Procedure 

#### 4.2.1. Apparatus

Conditioning was performed in a three-compartment apparatus (64 cm wide × 32 cm deep × 31 cm high) made of unplasticized polyvinyl chloride. The middle (neutral) compartment (10 × 30 × 30 cm) had white walls and a white floor. Two doors connected the middle compartment to the two conditioning compartments (25 × 30 × 30 cm each), with walls displaying either vertical or horizontal black-and-white stripes of the same overall brightness, and stainless-steel floors containing either 168 holes (diameter 0.5 cm) or 56 slits (4.2 × 0.2 cm each). 

The course of the animal was recorded with a video camera placed above the apparatus and analyzed with the ANY-maze Video Tracking Software (Stoelting Europe, Dublin, Ireland). The distance traveled and the time the animals spent in the compartments during each session were assessed. After each session, the CPP apparatus was cleaned with 70% camphorated ethanol solution. 

#### 4.2.2. Acquisition of Place Preference

The acquisition protocol comprised a pretest session on the first day, in which the animals were allowed to freely explore the three compartments. The pre-test was followed by four consecutive conditioning days, with two sessions per day separated by at least 4 h, and with a total of four conditionings for each stimulus. For cocaine and social interaction CPP groups, the less preferred compartment in the pretest was paired with the stimulus in the conditioning sessions. Cocaine or saline were injected intraperitoneally (i.p.) immediately before placing the rat into the stimulus-associated compartment. Cocaine (hydrochloride salt) (Gatt-Koller, Absam, Austria) was dissolved to a concentration of 15 mg/kg of pure cocaine base in a volume of 1 mL/kg of saline solution. The saline CPP group received saline in both compartments. In the social interaction conditioning sessions, each rat received an i.p. injection of saline and was placed in the associated compartment together with a conspecific of the same weight and gender, which was assigned in the first conditioning and remained the same for the whole duration of the experiment. On the sixth day, animals were tested for place preference by being placed in the middle compartment of the apparatus and allowed to move freely between all the compartments. All sessions—pre-test, conditionings, and CPP test—were of equal duration, i.e., 15 min. The preference score was calculated as the time spent in the stimulus-associated compartment in the test subtracted by the time spent in the same compartment in the pretest. For the saline control group, the preference score was calculated as the time spent during the test minus the pre-test in the pre-determined less preferred compartment. For social interaction CPP, time in the social interaction compartment during the test was shown. 

#### 4.2.3. Concurrent CPP Paradigm

Animals were simultaneously trained for CPP by pairing cocaine with one compartment and social interaction with the other compartment. The protocol comprised a pre-test session on day one, followed by four conditioning days with two sessions per day—one for each stimulus (social interaction session performed in the morning and cocaine session in the afternoon), and a CPP test on the day after the last conditioning session. All sessions were of equal duration (15 min). Results were expressed as a cocaine preference score, calculated as the time spent in the cocaine-associated compartment in the test minus the time spent in the same compartment in the pretest. As “touch” was reported as the major rewarding sensory component of the composite stimulus “social interaction” [12], time in social interaction, during which the social paired rats were “touching”, was evaluated in the first (C1) and last (C4) social conditioning session. 

#### 4.2.4. Persistence of Social Interaction/Cocaine CPP

To test whether social interaction and cocaine CPP is maintained after one and two weeks, a conditioned place preference protocol was performed, followed by two test sessions: Seven and fourteen days after the first test, in which the animals freely explored the three compartments for 15 min. Results were expressed as time spent in the social interaction- or cocaine-associated compartment subtracted from the time spent in the saline-associated compartment during the test for each time point (pretest, test day 1, test day 7, and test day 14).

### 4.3. Incorrect Transitions of Cephalocaudal Grooming Progression

The incorrect transition of cephalocaudal grooming pattern in rodents can be used as a stress marker [35]. Briefly, an analysis of the incorrect transitions in cephalocaudal grooming patterns during the CPP test was performed as previously described [17], in accordance with the following grooming stages: No grooming (0), paw licking (1), nose/face/head wash (2), body grooming (3), leg licking (4), and tail/genitals grooming (5). Correct transitions between grooming stages include the progressive transitions from one stage to the following one. Incorrect transitions pattern consisted of aborted, prematurely terminated (e.g., 3–0 or 4–0), skipped (e.g., 1–5 or 2–5), reversed (e.g., 3–2, 4–1 or 5–2), or incorrectly initiated (e.g., 0–4 or 0–5). Results were presented as the percentage of incorrect cephalocaudal grooming transitions.

### 4.4. Surgeries and Intra-NAc Infusions

**Figure 7:**
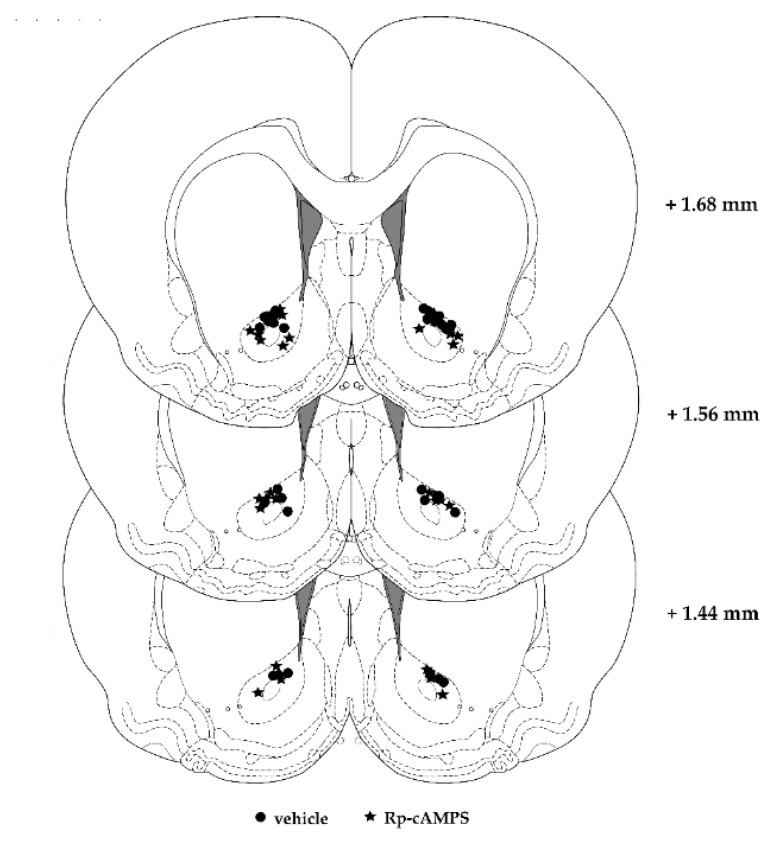
Representative coronal sections of the nucleus accumbens, with infusion sites of vehicle (circle) and Rp-cAMPS (star). The values shown on the right side of the coronal sections are the “distance from bregma”.

Rats were 7 weeks-old when subjected to surgery. Briefly, guide cannulae (Plastics One, 23G, Bilaney, Düsseldorf, Germany) were implanted bilaterally in the NAc region (anteroposterior: ±1.6 mm, mediolateral: ±2.3 mm, dorsoventral: −7.2 mm) (Figure 7), relative to bregma for infusion of the PKA inhibitor or vehicle following the procedure described in [44]. Two stainless steel screws were placed on the skull to provide additional stability, and cannulae and screws were held in place with a layer of dental cement. Dummy cannulae were placed in each guide to prevent blockage. Following surgery, the animals received postoperative analgesia and were allowed to recover for at least five days before the behavioral experiments. 

Rp-cAMPS (Tocris, Abingdon, United Kingdom) was dissolved in 0.9% sterile saline to a concentration of 40 µg/µL [2]. On the test day, animals received an infusion of 0.5 µL/side of vehicle (0.9% sterile saline solution) or Rp-cAMPS (20 µg/0.5 µL/side), over a period of 2 min. To prevent backflow, infusion cannulae remained in place for 3 min after the infusion. Animals were placed back in their home cages and, 30 min [9] after the infusion, were tested for CPP. 

### 4.5. Western Blot

The rats were euthanized 20 min after the end of the CPP test by an overdose of CO_2_ inhalation. The brains were removed and immediately frozen in isopentane in dry ice (−40 °C). Tissue punches from the NAc were collected from thaw-mounted 150 µm coronal sections obtained in a cryostat using a using a sample corer (Fine science tools, 11G–17G, Heidelberg, Germany) and frozen at −80 °C until used. In experiments where cannulae were implanted in the NAc for the infusion of the PKA inhibitor, cannulae placement was confirmed during this step, resulting in the exclusion of a total of two animals due to incorrect placement.

Total protein was extracted from the collected NAc tissue. Briefly, brain tissue was homogenized in a potter with RIPA buffer (Thermo Fisher Scientific, Vienna, Austria) supplemented with protease and phosphatase inhibitors (100×) (Thermo Fisher Scientific, Vienna, Austria). Samples were placed in an orbital mixer for 30 min at 4 °C and centrifuged for 15 min at 13,000× *g*, at 4 °C. The supernatant was collected and the protein concentration determined using a Bradford protein assay. Total extracts were stored at −80°C until further use.

Protein samples (20 µg) were prepared for SDS-polyacrylamide gel electrophoresis by adding Roti-Load buffer (Lactan, Graz, Austria), loaded onto 10% acrylamide gels and then transferred to PVDF membranes. Blots were blocked for 1 h at room temperature (RT) with either 5% non-fat dry milk (NFDM) or 5% bovine serum albumin (BSA)—for non-phosphorylated and phosphorylated proteins, respectively, in a 0.1% Tween 20 in Tris-buffered saline (TBS-T) solution. Membranes were incubated with primary antibodies, raised in rabbit or chicken, diluted in 5% NFDM/BSA in TBS-T, overnight, at 4 °C: CREB (1:1000, Cell Signaling Technology Europe (CST) #9197), pCREB (Ser133) (1:1000, Millipore #06-519), PKA (1:1000, Abcam #ab211265), pPKA (T197) (1:10000, Abcam #ab75991), DARPP-32 (1:1000, CST #2306), pDARPP-32 (Thr34) (1:500, CST #12438), and β-III Tubulin (1:50,000, Novus Biologicals #NB100-1612), used as loading control. Blots were washed 3 times for 10 min with 1% NFDM/1% BSA in TBS-T and incubated with the suitable anti-rabbit/chicken IgG horseradish peroxidase-conjugated secondary antibodies diluted in the same solution (1:20,000), for 1 h at RT. Again, blots were washed 3 times for 10 min and developed in a Chemidoc Imaging Sytem (Bio-Rad) after incubation with an enhanced chemiluminescence substrate (Bio-Rad, Vienna, Austria). Results were expressed as relative intensity of the ratio between non-phosphorylated and total protein, that were previously normalized to tubulin. Image Lab Software (Bio-Rad) was used to quantify the bands intensity. 

### 4.6. Statistical Analyses 

Statistical analyses were performed using GraphPad PRISM (GraphPad Software; CA, USA). All data were expressed as mean ± standard error of the mean (SEM), and *p* values < 0.05 were considered statistically significant. The significance between two experimental groups was tested using either a two-tailed unpaired Student´s *t*-test or a two-tailed unpaired Welch´s *t*-test for groups with significantly different variances. To test the statistical difference between three or more experimental groups, one or two-way analysis of variance (ANOVA) was used, depending on the number of variables to analyze, followed by a Newman-Keuls multiple comparisons post-hoc test. ANOVA with repeated measures were used when the samples were correlated. Cohen´s d was calculated to evaluate effect sizes. The Pearson correlation coefficient (r) was calculated to assess linear correlations between two variables of the same experimental group.

## Data Availability

The data presented in this study should be publicly available and cited in accordance with journal guidelines.

## Abbreviations

ANOVA	analysis of variance
BSA	bovine serum albumin
CA	conditioned activity
cAMP	cyclic adenosine monophosphate
CPP	conditioned place preference
CREB	cAMP response element-binding protein
DARPP-32	dopamine-regulated phosphoprotein-32
i.p.	intraperitoneal
Icv	intracerebroventricular
NAc	nucleus accumbens
NFDM	non-fat dry milk
pCREB	phosphorylated cAMP response element-binding protein
pDARPP-32	phosphorylated dopamine-regulated phosphoprotein-32
PKA	protein kinase A: cAMP-dependent protein kinase
pPKA	phosphorylated protein kinase A
RIPA	radioimmunoprecipitation assay
RT	room temperature
SI	social interaction
TBST-T	tris-buffered saline with Tween 20

## References

[1] Kelley A.E. (2004). Memory and addiction: Shared neural circuitry and molecular mechanisms. Neuron.

[2] Gerdjikov T.V., Giles A.C., Swain S.N., Beninger R.J. (2007). Nucleus accumbens PKA inhibition blocks acquisition but enhances expression of amphetamine-produced conditioned activity in rats. Psychopharmacology.

[3] Beninger R.J., Nakonechny P.L., Savina I. (2003). cAMP-dependent protein kinase and reward-related learning: Intra-accumbens Rp-cAMPS blocks amphetamine-produced place conditioning in rats. Psychopharmacology.

[4] Beninger R.J., Gerdjikov T. (2004). The role of signaling molecules in reward-related incentive learning. Neurotox. Res..

[5] Cervo L., Mukherjee S., Bertaglia A., Samanin R. (1997). Protein kinases A and C are involved in the mechanisms underlying consolidation of cocaine place conditioning. Brain Res..

[6] Sutton M.A., McGibney K., Beninger R.J. (2000). Conditioned locomotion in rats following amphetamine infusion into the nucleus accumbens: Blockade by coincident inhibition of protein kinase A. Behav. Pharmacol..

[7] Miserendino M.J.D., Nestler E.J. (1995). Behavioral sensitization to cocaine: Modulation by the cyclic AMP system in the nucleus accumbens. Brain Res..

[8] Lynch W.J., Taylor J.R. (2005). Persistent changes in motivation to self-administer cocaine following modulation of cyclic AMP-dependent protein kinase A (PKA) activity in the nucleus accumbens. Eur. J. Neurosci..

[9] Self D.W., Genova L.M., Hope B.T., Barnhart W.J., Spencer J.J., Nestler E.J. (1998). Involvement of cAMP-dependent protein kinase in the nucleus accumbens in cocaine self-administration and relapse of cocaine-seeking behavior. J. Neurosci..

[10] El Rawas R., Saria A. (2016). The Two Faces of Social Interaction Reward in Animal Models of Drug Dependence. Neurochem. Res..

[11] EL Rawas R., Amaral I.M., Hofer A. (2020). Social interaction reward: A resilience approach to overcome vulnerability to drugs of abuse. Eur. Neuropsychopharmacol..

[12] Kummer K., Klement S., Eggart V., Mayr M.J., Saria A., Zernig G. (2011). Conditioned place preference for social interaction in rats: Contribution of sensory components. Front. Behav. Neurosci..

[13] Peartree N.A., Hood L.E., Thiel K.J., Sanabria F., Pentkowski N.S., Chandler K.N., Neisewander J.L. (2012). Limited physical contact through a mesh barrier is sufficient for social reward-conditioned place preference in adolescent male rats. Physiol. Behav..

[14] Fritz M., El Rawas R., Salti A., Klement S., Bardo M.T., Kemmler G., Dechant G., Saria A., Zernig G. (2011). Reversal of cocaine-conditioned place preference and mesocorticolimbic Zif268 expression by social interaction in rats. Addict. Biol..

[15] Bregolin T., Pinheiro B.S., El Rawas R., Zernig G. (2017). Preventive strength of dyadic social interaction against reacquisition/reexpression of cocaine conditioned place preference. Front. Behav. Neurosci..

[16] Ribeiro Do Couto B., Aguilar M.A., Lluch J., Rodríguez-Arias M., Miñarro J. (2009). Social experiences affect reinstatement of cocaine-induced place preference in mice. Psychopharmacology.

[17] Lemos C., Salti A., Amaral I.M., Fontebasso V., Singewald N., Dechant G., Hofer A., El Rawas R. (2020). Social interaction reward in rats has anti-stress effects. Addict. Biol..

[18] Venniro M., Zhang M., Caprioli D., Hoots J.K., Golden S.A., Heins C., Morales M., Epstein D.H., Shaham Y. (2018). Volitional social interaction prevents drug addiction in rat models. Nat. Neurosci..

[19] El Rawas R., Klement S., Kummer K.K., Fritz M., Dechant G., Saria A., Zernig G. (2012). Brain regions associated with the acquisition of conditioned place preference for cocaine vs. social interaction. Front. Behav. Neurosci..

[20] Olsen C.M. (2011). Natural rewards, neuroplasticity, and non-drug addictions. Neuropharmacology.

[21] Been L.E., Hedges V.L., Vialou V., Nestler E.J., Meisel R.L. (2013). ΔJunD overexpression in the nucleus accumbens prevents sexual reward in female Syrian hamsters. Genes Brain Behav..

[22] Pitchers K.K., Vialou V., Nestler E.J., Laviolette S.R., Lehman M.N., Coolen L.M. (2013). Natural and drug rewards act on common neural plasticity mechanisms with ΔFosB as a key mediator. J. Neurosci..

[23] Reichel C.M., Wilkinson J.L., Bevins R.A. (2010). Reference place conditioning procedure with cocaine: Increased sensitivity for measuring associatively motivated choice behavior in rats. Behav. Pharmacol..

[24] Fritz M., El Rawas R., Klement S., Kummer K., Mayr M.J., Eggart V., Salti A., Bardo M.T., Saria A., Zernig G. (2011). Differential effects of accumbens core vs. shell lesions in a rat concurrent conditioned place preference paradigm for cocaine vs. social interaction. PLoS ONE.

[25] Lu L., Grimm J.W., Shaham Y., Hope B.T. (2003). Molecular neuroadaptations in the accumbens and ventral tegmental area during the first 90 days of forced abstinence from cocaine self-administration in rats. J. Neurochem..

[26] Terwilliger R.Z., Beitner-Johnson D., Sevarino K.A., Crain S.M., Nestler E.J. (1991). A general role for adaptations in G-proteins and the cyclic AMP system in mediating the chronic actions of morphine and cocaine on neuronal function. Brain Res..

[27] Freeman W.M., Nader M.A., Nader S.H., Robertson D.J., Gioia L., Mitchell S.M., Daunais J.B., Porrino L.J., Friedman D.P., Vrana K.E. (2001). Chronic cocaine-mediated changes in non-human primate nucleus accumbens gene expression. J. Neurochem..

[28] Hope B.T., Crombag H.S., Jedynak J.P., Wise R.A. (2005). Neuroadaptations of total levels of adenylate cyclase, protein kinase A, tyrosine hydroxylase, cdk5 and neurofilaments in the nucleus accumbens and ventral tegmental area do not correlate with expression of sensitized or tolerant locomotor responses to cocaine. J. Neurochem..

[29] Unterwald E.M., Fillmore J., Kreek M.J. (1996). Chronic repeated cocaine administration increases dopamine D1 receptor-mediated signal transduction. Eur. J. Pharmacol..

[30] Crawford C.A., Choi F.Y., Kohutek J.L., Yoshida S.T., McDougall S.A. (2004). Changes in PKA Activity and Gsα and Golfα Levels after Amphetamine and Cocaine-Induced Behavioral Sensitization. Synapse.

[31] Baldwin A.E., Sadeghian K., Holahan M.R., Kelley A.E. (2002). Appetitive instrumental learning is impaired by inhibition of cAMP-dependent protein kinase within the nucleus accumbens. Neurobiol. Learn. Mem..

[32] Wang L., Guo T., Guo Y., Xu Y. (2020). Asiaticoside produces an antidepressant—Like effect in a chronic unpredictable mild stress model of depression in mice, involving reversion of inflammation and the PKA/pCREB/BDNF signaling pathway. Mol. Med. Rep..

[33] Dwivedi Y., Mondal A.C., Shukla P.K., Rizavi H.S., Lyons J. (2004). Altered protein kinase A in brain of learned helpless rats: Effects of acute and repeated stress. Biol. Psychiatry.

[34] Logrip M.L., Koob G.F., Zorrilla E.P. (2011). Role of corticotropin-releasing factor in drug addiction: Potential for pharmacological intervention. CNS Drugs.

[35] Kalueff A.V., Tuohimaa P. (2004). Grooming analysis algorithm for neurobehavioural stress research. Brain Res. Protoc..

[36] Kummer K.K., Hofhansel L., Barwitz C.M., Schardl A., Prast J.M., Salti A., El Rawas R., Zernig G. (2014). Differences in social interaction vs. cocaine reward in mouse vs. rat. Front. Behav. Neurosci..

[37] Di Chiara G. (2002). Nucleus accumbens shell and core dopamine: Differential role in behavior and addiction. Behav. Brain Res..

[38] Ikemoto S., Qin M., Liu Z.-H. (2005). The functional divide for primary reinforcement of D-amphetamine lies between the medial and lateral ventral striatum: Is the division of the accumbens core, shell, and olfactory tubercle valid?. J. Neurosci..

[39] Corbit L.H., Fischbach S.C., Janak P.H. (2016). Nucleus accumbens core and shell are differentially involved in general and outcome-specific forms of Pavlovian-instrumental transfer with alcohol and sucrose rewards. Eur. J. Neurosci..

[40] Floresco S.B., Montes D.R., Tse M.M.T., van Holstein M. (2018). Differential contributions of nucleus accumbens subregions to cue-guided risk/reward decision making and implementation of conditional rules. J. Neurosci..

[41] Misra K., Pandey S.C. (2006). The decreased cyclic-AMP dependent-protein kinase A function in the nucleus accumbens: A role in alcohol drinking but not in anxiety-like behaviors in rats. Neuropsychopharmacology.

[42] Carr G.D., Phillips A.G., Fibiger H.C. (1988). Independence of amphetamine reward from locomotor stimulation demonstrated by conditioned place preference. Psychopharmacology.

[43] Solinas M., Chauvet C., Thiriet N., El Rawas R., Jaber M. (2008). Reversal of cocaine addiction by environmental enrichment. Proc. Natl. Acad. Sci. USA.

[44] Miller C.A., Marshall J.F. (2005). Molecular substrates for retrieval and reconsolidation of cocaine-associated contextual memory. Neuron.

